# Applying RE-AIM to Evaluate the External Validity of Weight Gain Prevention Interventions in Young Adults: A Systematic Review

**DOI:** 10.1097/PHH.0000000000001159

**Published:** 2020-04-17

**Authors:** Debra Haire-Joshu, Alexandra B. Morshed, Allison Phad, Shelly Johnston, Rachel G. Tabak

**Affiliations:** Center for Diabetes Translation Research (Drs Haire-Joshu and Tabak and Mss Phad and Johnston), Center for Obesity Prevention and Policy Research (Dr Haire-Joshu), and Prevention Research Center (Drs Morshed and Tabak), the Brown School, Washington University in St Louis, St Louis, Missouri.

**Keywords:** external validity, RE-AIM framework, weight gain prevention, young adults

## Abstract

The purpose of this review was to use RE-AIM (Reach, Effectiveness, Adoption, Implementation, Maintenance) to assess the extent to which weight gain prevention studies targeting young adults reported on elements of external validity.

**Design:**

Systematic review.

**Eligibility Criteria:**

Articles of interest included a lifestyle/behavioral intervention targeting weight gain prevention. Eligibility criteria included the following: study design of randomized controlled trials, quasi-randomized control trials, or natural experiments; average participant age between 18 and 35 years; study duration of at least 12 months; and published in English between January 2008 and May 2018. Studies had to report weight or body mass index as a measured outcome and were excluded if they were paired with smoking cessation programs, were conducted in specific groups (ie, pregnant women, breast cancer survivors), or were follow-ups to weight loss studies.

**Study Selection:**

After removing duplicates, the search yielded 11426 articles. Titles and abstracts were screened by 1 reviewer; 144 articles were assessed in a full-text review by 2 reviewers. Discrepancies were resolved by consensus. Nine studies (13 articles) were included in the review.

**Main Outcomes Measure(s):**

Reported elements of the RE-AIM framework.

**Results:**

A total of 9 studies met the selection criteria. All studies lacked full reporting on external validity elements. Of the total of 60 RE-AIM reporting criteria, 8 were reported by all 9 studies, 26 criteria were reported by fewer than 4 studies, and 22 criteria were not reported by any of the studies.

**Discussion:**

There remains inadequate reporting of elements of external validity and generalizability in weight gain prevention studies. This is a significant scientific constraint that limits the information required to disseminate and implement prevention of weight gain interventions for population impact. Standardized reporting may be needed to ensure results that demonstrate not only internal validity but also external validity and generalizability are needed to promote public health impact.

## Abstract 2

Supplemental Digital Content is Available in the Text.

Young adulthood is a period of high risk for excessive weight gain and development of obesity, representing an important target of intervention. On average, US adults gain 0.5 to 1.0 kg per year, resulting in an average 13-kg weight gain from early to middle adulthood.^1^,^2^ This average annual weight gain doubles the prevalence of obesity in the population, as young adults advance rapidly toward overweight or obesity by middle age,^2^ and is leading to a decrease in life expectancy in the United States. Woolf and Schoomaker^3^ reported that, between 1999 and 2017, age-adjusted midlife mortality rates for obesity increased by 114.0% (from 1.3 deaths/100000 to 2.7 deaths/100000). Midlife is a particularly susceptible period for young women, due to excess weight gain and retention during pregnancy and postpartum, and further increases the disparities among racial subgroups.^4^ Risk for excessive weight gain in early adulthood is also associated with early onset of diabetes, cardiovascular, and related chronic diseases, impacting quality of life and health care costs.^2^,^5^

Early weight gain prevention trials (1985-2011) were generally designed to test whether the intervention was or was not efficacious. Consistent with this focus on standard reporting elements for internal validity, reports of study findings primarily describe efficacy.^6^,^7^ Brought on by a lack of external validity, calls were made for more generalizable studies and the reporting on elements of external validity. External validity incorporates a better understanding of the generalizability of interventions across different populations, settings, and variations in treatment,^8^ which is needed to assess how well the research translates into practice.^9^ Reviews have recommended improvements in reporting on external validity components that influence dissemination and scale-up of interventions aimed at preventing obesity among this high-risk age group.^10^ Going beyond reporting, research with more relevance and generalizability is needed to impact chronic disease burden at a population level.^11^,^12^ To understand the gaps in external validity, it is important to review the extent to which current studies report generalizable findings. It is unclear whether weight gain prevention studies conducted since these prior reviews, and during times when calls for attention to balanced reporting were more prominent, have comprehensively addressed elements of both internal and external validity in describing their findings.

There are several approaches to guide and assess the balance of internal and external validity in study planning, execution, and reporting of study findings.^13^–^17^ The RE-AIM (Reach, Effectiveness, Adoption, Implementation, Maintenance) planning and evaluation framework^18^ guides the reporting of essential program elements addressing external validity that may improve the sustainable adoption and implementation of effective, generalizable, and evidence-based interventions. It has been used extensively over the past 2 decades in public health and health behavior change research to report on contextual factors related to external validity of interventions.^15^,^19^ The purpose of this review was to use RE-AIM to assess the extent to which weight gain prevention studies targeting young adults reported on elements of external validity.

## Methods

### Search strategy and study selection

We conducted a systematic literature review of weight gain prevention studies published in peer-reviewed journals in the 10-year period from January 2008 to May 2018. We chose to focus on studies publishing results during this time period because of the heightened attention to the importance of generalizability and expanded transparency in reporting external validity during this period. Databases searched included Scopus, Web of Science, EBSCOhost, and PubMed. A complete search strategy can be found in Supplemental Digital Content Appendix Table 1 (available at http://links.lww.com/JPHMP/A650). In summary, search terms were broad and included combinations, truncations, and synonyms of “weight,” “weight maintenance,” “young adult,” “lifestyle,” “behavioral,” and “intervention.” The search was limited to English. Additional studies were retrieved from reference lists of relevant studies; articles related to the included studies (ie, protocol papers, formative research) were also retrieved. Studies of interest included randomized controlled trials, quasi-randomized control trials, and natural experiments. Studies needed to include a behavioral or lifestyle intervention targeting weight gain prevention, a comparison group, and weight or body mass index (BMI) as a measured outcome. Excluded studies had an average participant age greater than 35 years or included interventions targeting pregnant women, paired weight gain prevention with smoking cessation programs, were conducted in specialized groups (ie, breast cancer survivors), or were follow-ups to weight loss studies. Since this review was concerned with annual weight gain, included studies had to be at least 12 months in duration, including length of intervention and follow-up. This review has been registered at PROSPERO (International Prospective Register of Ongoing Systematic Reviews, http://www.crd.york.ac.uk/prospero, CRD42018091824).^20^

After duplicates were removed, the initial search yielded 11426 studies (Figure). Titles and abstracts were screened by 1 reviewer (S.J.); 144 full-text studies were included for full-text review and assessed for inclusion by 2 reviewers (A.P. and S.J.). Discrepancies between reviewers were resolved by consensus. From these, 9 studies (from 13 articles) were eligible and included in the review.^21^–^33^

**FIGURE:**
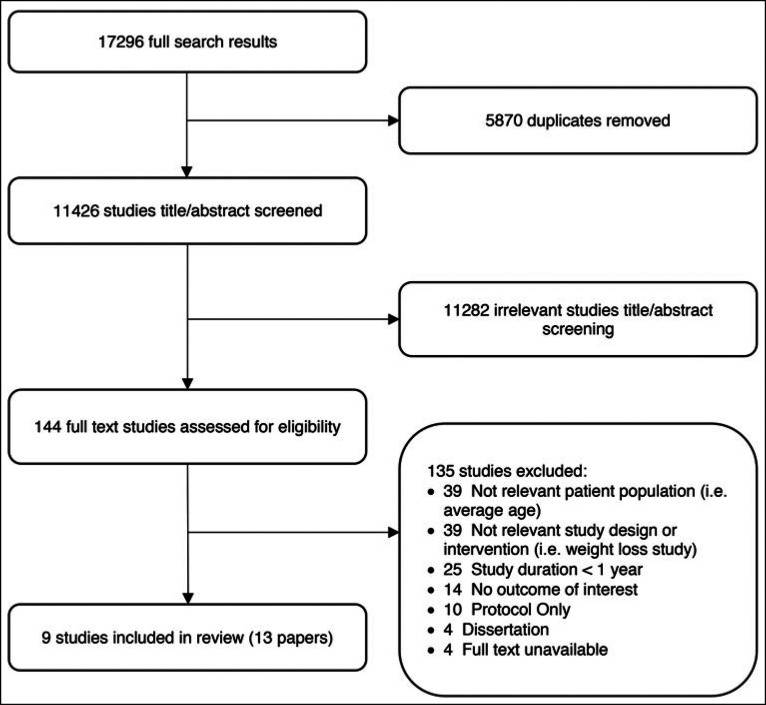
PRISMA Flow Chart—Process of Inclusion of Studies

### Data collection

Two reviewers (A.P. and A.G.) utilized the Cochrane data collection form to extract data from the 9 selected studies.^21^–^29^ The form was piloted on one study and then adjusted and used for those remaining. The following data were extracted: study details (authors, year, sponsorship source, country of publication), methods (design, aim of study/intervention, statistical methods, units of randomization/analysis), population characteristics (inclusion/exclusion criteria, baseline group differences, demographics), intervention details (setting, theoretical basis, content, providers, duration), and outcomes (weight, BMI, behavioral). Data were extracted for all articles by both reviewers, and discrepancies were resolved by consensus with the study team. Study screening and data extraction were completed using Covidence systematic review software.^34^

Following extraction, 2 reviewers (R.G.T. and A.M.) used the RE-AIM framework to assess the extent of reporting in the included studies on study elements related to internal and external validity and translation potential. The reviewers used the data extraction tool developed by Harden et al,^35^ designed specifically for conducting systematic reviews using RE-AIM. The tool measures multiple indicators for each RE-AIM element at multiple levels (ie, individual, provider, organizational): (1) reach (eg, description of target population), (2) efficacy/effectiveness (eg, use of intent to treat), (3) adoption (eg, method to identify setting, staff participation rate), (4) implementation (eg, timing and duration of contacts), and (5) maintenance (eg, program institutionalization). Both reviewers extracted data from all studies, and disagreements were resolved through discussion.

Two reviewers (A.P. and S.J.) assessed risk of bias using the Cochrane Collaboration's tool for assessing risk of bias.^36^,^37^ Domains of bias included selection, performance, detection, attrition, reporting, and other. Reviewers independently evaluated risk of bias, assigning “low risk of bias,” “high risk of bias,” or “unclear risk of bias” to each domain. Discrepancies were resolved by consensus.

## Results

Nine studies were included in the review.^21^–^29^ The studies were randomized controlled trials,^21^–^28^ with one cluster randomized controlled trial.^29^ The majority of trials were conducted in a college or university setting (n = 5)^21^,^24^–^27^ or a clinical setting (n = 3),^22^,^23^,^29^ with one conducted in the community surrounding a university.^28^ Seven studies were conducted in the United States,^21^,^22^,^24^–^28^ 1 study in the United Kingdom,^23^ and 1 study in Finland.^29^ In regard to intervention delivery, 3 studies included in-person interventions^26^,^28^,^29^ and 3 interventions solely utilized an online or other electronic platform.^21^,^24^,^25^ One study had the option of an in-person, online, or hybrid course.^27^ Two studies utilized in-person intervention delivery with electronic communication follow-up.^22^,^23^ Additional details are presented in Table 1.

**TABLE 1:** Weight Gain Prevention Study Details

Author (PublicationYear)	Baseline Characteristics, Age, Mean (SD), BMI, Mean (SD), Sample Size (n)	Setting, InterventionDelivery Method	Duration of Intervention, Follow-up	Weight Outcome, Change From Baseline to Follow up, kg^a^
Bertz et al (2015)^21^	Female: 51% White: 64% Age: 19 (0.4) BMI (C/I): 23.0 (3.1) kg/m^2^, 22.7 (2.9) kg/m^2^ n = 167, sites = 1	College campus Wi-Fi–enabled scales Web platform	I: 12 mo F: none	Mean (SD): C: 1.1 (4.4) I: −0.5 (3.7) *P* = **.035**
Biddle et al (2015)^23^	Female: 68.5% White: 80.2% Age: 32.8 (5.6) BMI: 34.6 (4.9) kg/m^2^ n = 187	UK primary care facilities In-person workshop Physical activity tracker Follow-up calls	I: 12 mo F: none	Mean (95% CI): C: −1.02 (−2.63, 0.58) I: −0.87 (−2.74, 1.00) *P* = .869
Greene et al (2012)^24^	Female: 63% White: 79% Age: 19.1 (1.1) BMI: 23.9 (4.1) kg/m^2^ n = 1689, sites = 8	College campuses Online platform, e-mail	I: 3 mo F: 15 mo	BMI,^b^ mean (SE): C: 23.5 (0.19) − 23.9 (0.20) kg/m^2^ I: 23.3 (0.20) − 23.5 (0.21) kg/m^2^ *P* > .05
Kattelmann et al (2014)^25^	Female: 67% White: 72.1% Age: 19.3 (1.1) BMI: 24.1 (4.4) kg/m^2^ n = 1639, sites = 1	College campus Online platform E-mail	I: 10 wk F: 15 mo	Mean (SD): C: 69.9 (16.2) − 70.6 (16.3) I: 68.6 (14) − 69.1 (13.8) *P* = .39
Katterman et al (2014)^26^	Female: 100% White: 62% Age, median (range): 22.3 (18-29) BMI: 26.63 kg/m^2^ n = 58, sites = 1	College campus In-person group meetings	I: 16 wk F: 12 mo	Estimated marginal means: C: +1.07 kg I: −2.24 kg *P* = **.008**
Lytle et al (2017)^27^	Female: 67.6% White: 72.6% Age: 22.7 (5.0) BMI: 25.4 (3.8) kg/m^2^ n = 441, sites = 3	Community college College course (in person, online, or hybrid)	I: 4 mo F: 24 mo	Mean (SD): C: 74.4 (0.863) I: 73.8 (0.857) *P* = .707
Metzgar and Nickols-Richardson (2016)^28^	Female: 100% White: 66% Age: 31.4 (8.1) BMI: 27.9 (6.8) kg/m^2^ n = 87	Community around college In-person group meetings	I: 12 mo F: none	Mean (SE): C: 77.9 (1.9) − 77.2 (2.2) RDs: 73.9 (1.6) − 75.2 (1.9) Counselor: 74.2 (1.1) − 75.1 (1.3) *P* > .05
Valve et al (2013)^29^	Female: 100% White: N/A Age, median (range): 19 (17-21) BMI: 22 (4.0) kg/m^2^ n = 1537, sites = 8	Vaccination centers (Finland) One-on-one counseling	I: 1.5-2.5 y F: none	BMI,^b^ median (IQR): I: 0.55 (1.59) C: 0.51 (1.75) *P* = .996
Wing et al (2016)^22^	Female: 78% White: 73% Age: 28.2 (4.4) BMI: 25.4 (2.6) kg/m^2^ n = 599, sites = 2	Clinical sites In-person group meetings, online refresher course, email	I: 4 mo F: 3 y (average)	Mean (SE): C: 0.26 (0.22) kg SC: −0.56 (0.22) LC: −2.37 (0.22) *P* (C vs SC) = **.018** *P* (C vs LC) **< .001** *P* (S vs LC) **< .001**

Abbreviations: BMI, body mass index; C, control; I, intervention; IQR, interquartile range; F, follow-up; LC, large changes; N/A, not available; RD, registered dietitian; SC, small changes.

^a^Boldface indicates statistical significance (*P* < .05).

^b^BMI reported as primary outcome, see the Supplemental Digital Content Appendix (available at: http://links.lww.com/JPHMP/A650) for additional details.

A summary of the RE-AIM results by each element is provided in Table 2; detailed results are available in Supplemental Digital Content Appendix Tables 2-4 (available at http://links.lww.com/JPHMP/A650). Harden et al^35^ included a total of 60 RE-AIM recommended reporting criteria. Of these, 8 criteria were reported by all 9 studies, 26 criteria were reported by 4 or fewer studies, and 22 criteria were not reported by any of the studies. The 8 reported criteria were consistent with those required by current CONSORT guidelines (ie, inclusion/exclusion criteria, attrition, and number, timing, duration of contacts).

**TABLE 2:** RE-AIM Criteria^28^ Included in Each Study

RE-AIMElement	Criteria	Study									Total
		Bertz et al (2015)^21^	Biddle et al (2015)^23^	Greene et al (2012)^24^	Kattelmann et al (2014)^25^	Katterman et al (2014)^26^	Lytle et al (2017)^27^	Metzgar and Nickols-Richardson (2016)^28^	Valve et al (2013)^29^	Wing et al (2016)^22^	
Reach	Described target population	x	x	x	x	x	x	x	x	x	9
	Demographic, behavioral information about target population				x						1
	Method to identify the target population		x								1
	Recruitment strategies	x	x	x	x	x	x	x	x	x	9
	Inclusion/exclusion criteria for individuals	x	x	x	x	x	x	x	x	x	9
	Eligible, invited (exposed to recruitment) potential participants								x		1
	Sample size	x	x	x	x	x	x	x	x	x	9
	Individual participation rate (sample size/eligible invited potential participants)								x		1
	Comparisons between the target population and the study sample				x		x				2
	Statistical comparisons between the target population and the study sample										0
	Cost of recruitment										0
	Qualitative methods to measure reach										0
Effectiveness	Report of mediators					x					1
	Report of moderators	x		x	x					x	4
	Intent-to-treat	x	x					x	x	x	5
	Imputation procedures		x				x			x	3
	Quality-of-life measures		x							x	2
	Unintended consequences measures/results	x		x						x	3
	Percent attrition (at program completion)	x	x	x	x	x	x	x	x	x	9
	Cost-effectiveness										0
	Qualitative methods to measure efficacy/effectiveness										0
Adoption,	Eligible, invited potential settings						x				1
setting	Number of participating settings			x	x		x		x	x	5
	Setting participation rate						x				1
	Description of the targeted location						x				1
	Inclusion/exclusion criteria of the setting						x				1
	Description of intervention location	x		x	x	x	x		x		6
	Method to identify the setting						x				1
	Comparisons between the targeted and participating settings										0
	Statistical comparisons between the targeted and participating settings										0
	Average number of persons served per setting						x				1
Adoption,	Eligible, invited potential providers (staff)										0
provider	Number of participating providers (staff)							x			1
	Provider (staff) participation rate										0
	Method to identify target providers										0
	Level of expertise of providers					x		x	x	x	4
	Inclusion/exclusion criteria for providers										0
	Comparisons between targeted and participating providers (staff)										0
	Statistical comparisons between targeted and participating providers (staff)										0
	Measures of cost adoption										0
	Dissemination beyond originally planned										0
	Qualitative methods to measure adoption										0
Implementation	Theory-based		x	x	x	x	x	x	x	x	8
	Number of intervention contacts	x	x	x	x	x	x	x	x	x	9
	Timing of intervention contacts	x	x	x	x	x	x	x	x	x	9
	Duration of intervention contacts			x		x		x	x		4
	Extent protocol delivered as intended (fidelity)									x	1
	Consistency of implementation across settings or providers						x				1
	Participant attendance/completion rates	x	x	x	x	x	x	x	x	x	9
	Measure of intervention cost						x				1
	Qualitative methods to measure implementation							x			1
Maintenance, individual	Follow-up outcome measures at some duration after intervention termination		x	x		x					3
	Attrition/loss to follow-up of individuals		x	x		x					3
	Qualitative methods to measure individual maintenance of the intervention										0
Maintenance, organization	Intervention alignment with the organization's mission										0
	Maintenance of the program after completion of the study										0
	Modifications made to the original program										0
	Institutionalization of the program in the setting or system										0
	Attrition/loss to follow-up of settings						x				1
	Qualitative methods to measure organizational maintenance/sustainability										0

Reach was evaluated by 12 criteria including descriptions of who was intended to benefit (ie, the target population), who actually participated or was exposed to the intervention, how many persons participated out of those intended or targeted, and the characteristics of those who took part compared with those who did not.^15^ The target population was described by all studies but was most often limited in detail, including 2 characteristics (eg, female students, aged 18-30 years), making it difficult to determine a denominator for the percentage of the target population reached. Three studies only recruited women,^26^,^28^,^29^ while the other 6 studies included a majority of female participants (51%-78%).^21^–^25^,^27^ A majority of participants in all studies were white (62%-80%).^21^–^29^ Three of the 9 studies referenced attempts to address diversity/representation in their participant pool.^22^,^23^,^27^ Participants in 4 studies had an average baseline BMI in the normal weight range,^21^,^24^,^25^,^29^ participants in 4 studies fell in the overweight range,^22^,^26^–^28^ and 1 study had participants in the obese range.^23^ Three studies required a BMI above 18.5 kg/m^2^ to avoid participants falling below normal weight range.^22^,^26^,^27^ All studies reported recruitment strategies, inclusion/exclusion criteria for study participants, and sample size. Two studies described differences and similarities between the target and study populations.^25^,^27^ Only one study reported on participant eligibility and individual participation rate.^29^ None of the studies reported on recommended reach criteria or statistical comparisons between the target and study populations, cost of recruitment, or use of qualitative methods to measure reach or participation rates.

Effectiveness (or efficacy) was evaluated by 9 criteria including the degree to which the intervention changes health outcomes and quality of life, taking into account unintended or negative results.^15^ Six studies found the intervention had no effect on BMI outcomes between the control and intervention groups at follow-up.^23^–^25^,^27^–^29^ Three studies found statistically significant differences in change in weight or BMI between the intervention and control groups.^21^,^22^,^26^ Kattelmann et al^25^ included gender as a fixed effect in their model to account for different retention rates between males and females. Three studies 21,22,38 cited unintended consequences of the intervention including reduction below normal weight^24^ and rapid weight change associated with self-weighing.^21^ All studies addressed attrition; none of the studies addressed cost-effectiveness or qualitative measures of effectiveness.

Adoption was assessed at the setting and individual provider levels (by 10 and 11 criteria, respectively) including the number and proportion of settings and staff members who agreed to participate in delivering the intervention and how representative they were of the intended audience in terms of the setting and staff.^15^ One study reported 8 of the 10 setting criteria, allowing for calculation of the setting-level participation rate and reporting of the average number of persons served per participating location.^27^ Five studies reported on the number of participating sites,^22^,^24^,^25^,^27^,^29^ whereas 6 studies described intervention location.^21^,^24^–^27^,^29^ None of the studies reported on the criteria of comparisons between targeted and participating sites. With regard to adoption by providers, only 4 studies reported the level of expertise of the intervention providers^22^,^26^,^28^,^29^ or training^28^,^29^ and supervision of the intervention providers.^26^,^28^,^29^ The adoption or participation rate for individual providers was not calculable for any studies, since only one study reported the number of participating intervention staff or providers^28^ and none reported the number of eligible individual providers or their characteristics. Similarly, none of the studies reported differences between targeted and participating providers or statistical comparisons between these groups, cost of adoption, dissemination of the intervention beyond where originally planned, or use of qualitative methods to measure individual provider adoption.

Implementation was assessed by the degree to which studies reported on 9 criteria including whether settings and staff members delivered the intervention as intended, the fidelity of intervention delivery, and costs.^15^ All studies reported individual participant engagement in terms of number, timing, duration of contact, and participant attendance. Only one study^22^ described whether the intervention protocol was delivered as intended, reporting that sessions sampled for measurement (20% of all sessions delivered) presented appropriate behavioral content. One study^28^ reported qualitative data regarding quality of intervention content delivery between individual providers; another^27^ included information about consistency of implementation across settings. Finally, one study^27^ reported partial costs, that is, tuition costs of the for-credit course made available to intervention group participants free of charge.

Maintenance was evaluated as the sustained effectiveness at the participant level (per 3 criteria), and the sustained delivery of the intervention at the setting or staff level (per 6 criteria), including the alignment of the intervention with organizational mission, objectives, and goals and integration into job descriptions and performance evaluations.^15^ At participant level, 3 studies included follow-up outcome measures after intervention termination, of which all reported 60% to 70% retention at follow-up and some of which differed by race/ethnicity, age, gender, and baseline BMI of study participants.^23^,^24^,^26^ At the setting level, only one study reported attrition of intervention sites at follow-up, reporting no sites lost to follow-up.^27^ None of the studies reported on any of the other criteria including qualitative methods to capture individual-level outcomes or maintenance of changes on whether the intervention was still in place after completion of the research study.

### Risk of bias

Using the Cochrane risk of bias tool,^37^ the 9 studies included in this review were overall rated low to unclear risk of bias (see Supplemental Digital Content Appendix Table 5, available at http://links.lww.com/JPHMP/A650). The greatest source of bias from the reviewed studies was performance bias, as blinding of participants and study personnel was often not conducive with the study design.^22^–^27^,^29^ One study^28^ blinded study participants to group assignment and it was unclear whether a second study^21^ blinded participants. Two studies had a high risk of attrition bias due to a large volume of missing follow-up data^23^ and difference in BMI and desire to lose weight between completers and noncompleters.^24^ We assessed low risk of bias due to cluster randomized study design for Valve et al^29^; there was no recruitment bias, as clusters were randomized after recruitment and analysis was appropriate for a cluster design.^37^

## Discussion

The purpose of this review was to use RE-AIM to assess the extent to which weight gain prevention studies targeting young adults, and reporting results within the past decade, included elements of external validity. Our results suggest that there remains inadequate reporting on recent weight gain prevention studies with regard to components of external validity and generalizability.^39^ Issues critical to translating research findings to public health impact often receive little attention when compared with intervention efficacy in narrow research settings.^40^ This is a significant scientific constraint that limits the information required to disseminate and implement these interventions for population impact.^39^,^41^

This review offers several insights into the comprehensiveness of reporting by studies on weight gain prevention. First, there is an overall general lack of reporting by studies on all RE-AIM criteria. Of the total of 60 RE-AIM criteria,^35^ 37% (n = 22) were not reported by any studies and only 13% (n = 8) were reported by all studies. Despite the call for more comprehensive presentation of weight gain prevention study results, this dearth of reporting on elements of external validity shows that there has been minimal improvement in the past decade.^9^,^10^,^42^,^43^ The lack of information regarding external validity greatly limits interpretation and comparisons across studies that are required to fully understand impact and to inform future research efforts.^15^,^44^ Consistent reporting of external validity of weight gain prevention studies is needed to more effectively translate results into evidence-based policy and practice and to push the field to incorporate external validity into study planning and execution.

Second, the RE-AIM elements that were most often reported aligned with elements often required by journal or CONSORT publication guidelines.^45^ For example, of the 15 criteria for reach, all studies reported on the 4 criteria required by CONSORT guidelines.^45^ In contrast, 2 or fewer studies reported on the remaining 9 reach criteria such as enrollment, recruitment and participation rates, or costs of recruitment, which are rarely required for publication.^38^,^46^ Publication requirements appear to influence whether elements of external validity are, or are not, addressed. Glasgow and colleagues^16^ have proposed an expanded CONSORT figure to increase the transparency in reporting external validity. Given the adherence to current guidelines, requiring this expanded guideline has the potential to enhance reporting.

It is also important to note that only 3 of 9 studies reported a significant effect on the primary outcome of weight but that there was minimal information provided on external factors needed to understand the full scope, or lack thereof, of intervention effectiveness. Without detailed reporting on setting, provider, and participant adoption and fidelity, it is not possible to determine whether interventions found not to impact weight were not successful due to the intervention itself or due to implementation failure or lack of engagement. Consistent and comprehensive reporting is needed to inform the science of what and how interventions work, and who they work best with, to improve the development of future interventions.

Finally, there remains a dearth of reporting on differences between settings and providers who accept or decline to adopt an intervention.^16^ This makes it difficult to determine what criteria might be needed for a site to successfully deliver the intervention, who in a real-world setting is best suited to deliver the intervention, or what settings might be appropriate for translation. There were also significant gaps in how implementation or maintenance was reported in these studies, including how consistently an intervention was delivered, whether adaptations to the original intervention were made, and elements of intervention continuation.^41^ This makes it difficult to determine whether a weight gain prevention intervention can be effectively delivered, in what setting, by whom it can be delivered, and whether it is sustainable.^15^,^47^ These reporting omissions prevent the timely dissemination of interventions and contribute to the decades-long gap between research and real-world practice.^16^

In summary, to enhance the impact of weight gain prevention intervention studies on a population level, the emphasis on designing and executing studies to produce generalizability findings and the reporting of external validity elements must improve. Over the past decade, support for transparency in research^48^ has resulted in tools and checklists to aid in a balanced reporting process.^13^,^14^,^45^,^49^ Adoption of the expanded 2017 CONSORT criteria for nonpharmacologic clinical trials, which include both internal and external validity elements,^14^,^45^ also encourages consistent and balanced reporting.^16^ Standardizing requirements to include components of external validity, such as those proposed by Glasgow and colleagues,^16^ will improve the quality, comprehensiveness, and consistency of study reporting, necessary for the better interpretation and understanding of findings of current studies. Utilization of tools such as RE-AIM^41^ and PRECIS-2^17^ to help design weight gain prevention studies with a greater focus on external validity in addition to consistent reporting of external validity components of studies is needed to more effectively translate results into evidence-based policy and practice.

### Strengths and limitations

To our knowledge, there have been no other reviews of weight gain prevention interventions among young adults using the RE-AIM framework to address external validity. This review expands on recommendations from several prior reviews to address rigor and external validity of research related to annual weight gain as a critical obesity prevention target. In addition to careful abstraction of relevant studies by research staff, 2 expert reviewers further assessed studies using the RE-AIM tool. Limitations included the risk of bias due to study attrition and inability to compare outcomes across studies due to variation in reporting.

**Implications for Policy & Practice**

- *Practice*: Reporting on external validity is needed to determine whether a weight gain prevention intervention can be effectively delivered, in what setting, by whom it can be delivered, and whether it is sustainable in practice.
- *Policy*: Consistent reporting of external validity of weight gain prevention studies is needed to more effectively translate results into evidence-based policy and practice.
- *Research*: The lack of generalizable findings from studies designed to prioritize primarily internal validity and the lack of information regarding external validity greatly limit interpretation and comparisons across studies that are required to fully understand impact and to translate research to practice.

## Conclusion

Prevention of weight gain in young adults is critical to reversing the obesity epidemic.^4^ Despite a heightened focus on balanced reporting of study validity, there remains inadequate reporting of prevention of weight gain studies with regard to elements of external validity and generalizability. The continued lack of prioritizing generalizability in study design and execution and reporting on dimensions of external validity is a significant scientific constraint that limits opportunities to disseminate and implement prevention of weight gain interventions for population impact. Standardized reporting may be needed to ensure results that demonstrate not only internal validity^16^ but also external validity and generalizability are needed to promote public health impact.^15^

## Supplementary Material

SUPPLEMENTARY MATERIAL
