# Supplementary material for: Applying RE-AIM to Evaluate the External Validity of Weight Gain Prevention Interventions in Young Adults: A Systematic Review
Source: J Public Health Manag Pract. 2020 Apr 17;27(2):154–65. doi: 10.1097/PHH.0000000000001159 (PMC7837750; doi:10.1097/PHH.0000000000001159)
Supplement: SUPPLEMENTARY MATERIAL [file jpump-27-154-s001.docx]

Appendix Table 1: Search Details

| **Database** | **Search** |
| --- | --- |
| Scopus | ( ( ( TITLE-ABS-KEY ( young W/2 ( adult OR person OR people ) ) AND NOT KEY ( child OR children OR pediatri* ) ) ) AND ( ( TITLE-ABS-KEY ( weight W/2 ( gain* OR maintenance OR maintai* OR chang* OR los* OR reduc* OR increas* OR control ) ) OR TITLE-ABS-KEY ( "body mass index" OR bmi ) OR TITLE-ABS-KEY ( obes* OR overweight ) OR TITLE-ABS-KEY ( anthropometr* ) ) ) AND ( TITLE-ABS-KEY ( ( ( lifestyle OR "life style" OR behavio* ) OR ( diet* OR nutri* OR exercise OR activ* OR "physical training" OR spor* ) ) W/5 ( intervention OR program OR strategy OR trial ) ) ) ) AND ( LANGUAGE ( english ) ) AND ( LIMIT-TO ( PUBYEAR , 2018 ) OR LIMIT-TO ( PUBYEAR , 2017 ) OR LIMIT-TO ( PUBYEAR , 2016 ) OR LIMIT-TO ( PUBYEAR , 2015 ) OR LIMIT-TO ( PUBYEAR , 2014 ) OR LIMIT-TO ( PUBYEAR , 2013 ) OR LIMIT-TO ( PUBYEAR , 2012 ) OR LIMIT-TO ( PUBYEAR , 2011 ) OR LIMIT-TO ( PUBYEAR , 2010 ) OR LIMIT-TO ( PUBYEAR , 2009 ) OR LIMIT-TO ( PUBYEAR , 2008 ) ) |
| Web of Science | TOPIC: ((weight NEAR/2 (gain* OR maintenance OR maintai* OR chang* OR los* OR reduc* OR increas* OR control)) OR “body mass index” OR BMI OR obes* OR overweight OR anthropometr*)  **AND** TOPIC: (young NEAR/2 (adult OR person OR people))  **AND** TOPIC: ((lifestyle OR “life style” OR behavio* OR diet* OR nutri* OR exercise OR activ* OR “physical training” OR spor*) NEAR/5 (intervention OR program OR strategy OR trial))  **AND** YEAR PUBLISHED: (2008-2018)  **AND** LANGUAGE: (English) |
| EBSCOhost:   - Academic Search Complete - Cinahl Plus - Family & Society Studies Worldwide - Global Health - MEDLINE - Psyc   ARTICLES   - PsycINFO | **(TI** ( weight N2 (gain* OR maintenance OR maintai* OR chang* OR los* OR reduc* OR increas* OR control) OR “body mass index” OR BMI OR obes* OR overweight OR anthropometr* ) **OR**  **SU** ( weight N2 (gain* OR maintenance OR maintai* OR chang* OR los* OR reduc* OR increas* OR control) OR “body mass index” OR BMI OR obes* OR overweight OR anthropometr* ) **OR**  **AB** ( weight N2 (gain* OR maintenance OR maintai* OR chang* OR los* OR reduc* OR increas* OR control) OR “body mass index” OR BMI OR obes* OR overweight OR anthropometr* ))  **AND** (**TI** ( young N2 (adult OR person OR people) ) **OR** **SU** ( young N2 (adult OR person OR people) ) **OR**  **AB** ( young N2 (adult OR person OR people) ) **NOT** **SU** ( child OR children OR pediatri* )) **AND**  (**TI** ((lifestyle OR “life style” OR behavio* OR diet* OR nutri* OR exercise OR activ* OR “physical training” OR spor*) N5 (intervention OR program OR strategy OR trial) ) **OR** **SU** ( (lifestyle OR “life style” OR behavio* OR diet* OR nutri* OR exercise OR activ* OR “physical training” OR spor*) N5 (intervention OR program OR strategy OR trial) ) **OR** **AB** ((lifestyle OR “life style” OR behavio* OR diet* OR nutri* OR exercise OR activ* OR “physical training” OR spor*) N5 (intervention OR program OR strategy OR trial))) **Limiters** - Published Date: 20080101-20181231 **Narrow by Language:**- english |
| PubMed | "young adult"[MeSH Terms] OR "young adult"[Other Term] **AND** diet[MeSH Terms] OR "diet therapy"[MeSH Terms] OR exercise[MeSH Terms] OR "exercise therapy"[MeSH Terms] OR sports[MeSH Terms] OR "life style"[MeSH Terms] OR "health behavior"[MeSH Terms] OR lifestyle[Other Term] OR “life style”[Other Term] OR behavio*[Other Term] OR diet*[Other Term] OR nutri*[Other Term] OR exercise[Other Term] OR activ*[Other Term] OR “physical training”[Other Term] OR spor*[Other Term] **AND** "body weight"[MeSH Terms] OR overweight[MeSH Terms] OR obesity[MeSH Terms] OR "body mass index"[MeSH Terms] OR weight[Other Term] OR “body mass index”[Other Term] OR BMI[Other Term] OR obes*[Other Term] OR overweight[Other Term] OR anthropometr*[Other Term] **AND** English[Language] **Filter** From 2008/01/01 to 2018/12/31 |
| Cochrane Library | (weight near/2 (gain* or maintenance or maintai* or chang* or los* or reduc* or increas* or control)) or "body mass index" or BMI or obes* or overweight or anthropometr*:ti,ab,kw (Word variations have been searched)  **AND** young near/2 (adult or person or people):ti,ab,kw not child or children or pediatri*:kw (Word variations have been searched) **AND** (lifestyle or "life style" or behavio* or diet* or nutri* or exercise or activ* or "physical training" or spor*) near/5 (intervention or program or strategy or trial):ti,ab,kw (Word variations have been searched) **AND** Publication Year from 2008 to 2018 |

Appendix Table 2: Reach and representation

| **First author, year** | **Target population description** | **Recruitment strategies** | **Individual inclusion/exclusion criteria** | **Baseline characteristics,**  **Age, mean (SD)**  **BMI, mean (SD)**  **Sample size (n)** |
| --- | --- | --- | --- | --- |
| Bertz 2015 | College students, freshmen | - class announcements - email listservs - flyers | Inclusion: 18–25 years, first-year college student, BMI ≥18.5 kg/m2  Exclusion: diabetes, pregnancy or planning to become pregnant during study, an eating disorder or history thereof, and a score of >45 on the drive for objective thinness questionnaire | - Female: 51% - White: 64% - Age: 19 (0.4) - BMI:   - C: 23.0 (3.1) kg/m^2^,   - I: 22.7 (2.9) kg/m^2^   n = 167, sites = 1 |
| Biddle 2015 | Adults with overweight (with an additional risk factor for T2DM) or obesity | - Electronic database search from general practitioner - Referral by general practitioner via invitation - Mass mail to patients attending local hospital | Inclusion: 18–40 years; BMI > 25.0, and with one or more additional risk factor for diabetes  Exclusion: Significant illness, steroid use, diabetes, pregnancy or an inability to communicate in English (From protocol paper) | - Female: 68.5% - White: 80.2% - Age: 32.8 (5.6) - BMI: 34.6 (4.9) kg/m^2^   n = 187, sites = n/a |
| Greene 2012 | College students, full- time | - flyers - online and class announcements - newspaper ads - table tents in dining halls | Inclusion: 18–24 years, full-time first-, second-, or third-year college student enrolled in one of the 8 participating universities  Exclusion: BMI <18.5 kg/m2; health conditions that might interfere with changes in diet and physical activity; pregnant, lactating or majoring in nutrition or exercise science | - Female: 63% - White: 79% - Age: 19.1 (1.1) - BMI: 23.9 (4.1) kg/m^2^   n = 1689, sites = 8 |
| Kattelmann 2014 | College students, full-time | - class announcements - emails - flyers - letters - resident housing meetings | Inclusion: 18–24 years, full-time first-, second-, or third-year college student with regular access to an Internet-connected computer  Exclusion: BMI<18.5 kg/m2; having a life-threatening condition such as pregnancy or other diet- and/or activity-related medical restriction; majoring in nutrition, exercise science, and/or health promotion; currently enrolled in a nutrition course | - Female: 67% - White: 72.1% - Age: 19.3 (1.1) - BMI: 24.1 (4.4) kg/m^2^   n = 1639, sites = 1 |
| Katterman 2014 | College students, female | - email listervs - flyers | Inclusion: female, undergraduate or graduate student (full- or part-time), BMI between 23- 30 kg/m2, planning to be in the Philadelphia >=1 year  Exclusion: BMI >32 kg/m2, current or or past eating disorder diagnosis, unable to attend any of the group sessions | - Female: 100% - White: 62% - Age, median (range): 22.3 (18-29) - BMI: 26.63 kg/m^2^   n = 58, sites = 1 |
| Lytle 2017 | Community college (2 yr) students, non-traditional | - class announcements - emails - flyers - information tables word-of-mouth | Inclusion: 18-35 years, BMI between 20-34.9 kg/m2, living in area >=2 years | - Female: 67.6% - White: 72.6% - Age: 22.7 (5.0) - BMI: 25.4 (3.8) kg/m^2^   n = 441, sites = 3 |
| Metzgar 2016 | Healthy premenopausal women, aged 18–45 y, with a body mass index (BMI) of >18.5 kg/m2 | - word-of-mouth - email - flyers, campus/community | Inclusion: 18-45 years, BMI>18.5 kg/m2, pre-menopausal, eumenorrhea,  Exclusion: amenorrhea; presented with depressive symptomology (score of >50 on the Zung Self-Rating Depression Scale/Status Inventory); health conditions that might interfere with changes in diet and physical activity; used supplements and/or medications that may influence BW regulation; bariatrics surgery; pregnant, lactating or planning to become pregnant | - Female: 100% - White: 66% - Age: 31.4 (8.1) - BMI: 27.9 (6.8) kg/m^2^   n = 87, sites = n/a |
| Valve 2013 | Females age 17-21 (Finland) | - From a population-based HPV vaccination trial | Inclusion: Finnish female aged 17–21 at the baseline of the present study and consenting to participate  Exclusion: pregnancy | - Female: 100% - White: N/A - Age, median (range): 19 (17-21) - BMI: 22 (4.0)kg/m^2^   n = 1537, sites = 8 |
| Wing 2016 | Young adults interested in weight gain prevention | - Emails - Mass mailings | Inclusion: 18–35, BMI of 21–30.9 kg/m2, ability to participate in the program (eg, Internet access, English speaking), ability to walk for activity  Exclusion: 10 pound weight loss in the past six months, bariatric surgery, hospitalization for or history of depression or psychiatric disorder (including eating disorders), past diagnosis or current symptoms of alcohol or substance abuse, pregnant lactating or planning to become pregnant | - Female: 78% - White: 73% - Age: 28.2 (4.4) - BMI: 25.4 (2.6)kg/m^2^   n = 599, sites = 2 |

Appendix 3: Adoption (setting and provider) and implementation

| **First author, year** | **Setting** | **Theoretical basis** | **Intervention description/**  **Comparator description** | **Intervention contacts, number, timing, and duration** | **Intervention / Follow-up duration** | **Extent protocol delivered as intended (fidelity)** | **Participant attendance/**  **completion rates** |
| --- | --- | --- | --- | --- | --- | --- | --- |
| Bertz 2015 | College campus | N/A | Intervention:  1) Video lecture  2) Intervention strategies:  - daily self-weighing - graphical feedback of the user’s weight plotted over time - email reminders after 3 missed days of self-weighing  Control:  1) Intro video:  - purpose of study,  - importance of maintaining weight through young adulthood  - strategies to maintain weight 2) Given Wi-Fi scales and told to weigh as frequently as they wanted | Provider: PI (initial lecture), Wi-Fi scale and web platform Number: 1 video lecture, median (IQR) of self-weighing 5 (2.1) and 5.8 (1.7) at 6 and 12 months Timing: daily feedback after self-weighing Duration: unknown | I: 12 months F: none | N/A | I: 95% participants weighed themselves ≥3 times/week and 67% weighed themselves ≥5times/week Median frequency (IQR) of self-weighing 5.8 (1.7) times/year at 1 year |
| Biddle 2015 | Primary care facilities (United Kingdom) | Adaptation of PREPARE and DESMOND structured education programs included: Bandura’s Social Cognitive Theory, Gollwitzer’s implementation intentions concept, Behavioral Choice Theory, Leventhal’s Common Sense Model | Intervention:  1) Education workshop:  - knowledge and perceptions of prevalent risk factors for type 2 diabetes and  - promote sedentary behavior change.  2) Self-monitoring of physical activity.  3) Follow up call to discuss goals and barriers to behavior change and regular text messages to encourage self-monitoring  Control:  1) Information leaflet containing:  - key illness perceptions of being at risk of T2DM - importance of increasing physical activity and decreasing sedentary behavior | Provider: Trained educators Number: 1 workshop, 1 follow-up call Timing: follow up at 6 weeks Duration: 3 hour workshop | I: 12 months  F: none | N/A | 24% of participants assigned to intervention did not attend workshop |
| Greene 2012 | College campus (8 sites) | Transtheoretical model, social cognitive model, Dick and Carey’s System of Instructional Design, Keller’s Instructional Motivational Model | Intervention:  1) Online educational program: - improved fruit and vegetable intake and  - sustained, enjoyable physical activity:  - eating competence  - appreciation of the value and work of the human body and accepting diversity in size and shape - improve attitudes, self-efficacy, and behaviors.  - Weekly goal setting.  - Weekly email reminders Control: none | Provider: online web portal Number: 10 lessons Timing: weekly Duration: 15 minutes | I: 3 months F: 15 months | N/A | 84% completed all 10 lessons, 5.1% did not complete any lessons Average time spent on lesson (among those completing) 7.8 minutes |
| Kattelmann 2014 | College campus | Precede-proceed process used to develop Dick and Carey's Model of Instructional Design, Transtheoretical Model of Behavior Change | Intervention:  1) Educational lessons: - eating behavior,  - physical activity,  - stress management, and  - healthy weight management  Email reminders: - reinforced behavior from lesson,  - visit web portal, view lessons, and set goals Control: none | Provider: online web portal  Number: 21 lessons, 30 email reminders Timing: first 10 weeks-2 lessons, 3 reminders per week plus 1 encouragement to view new lessons. After 10 weeks - 4 reminders/month Duration: 30 minute lessons | I: 10 weeks F: 15 months | N/A | 70% participants accessed lessons during intervention period 31% spent > 30 min viewing lesson |
| Katterman, S. 2014 | College campus | Acceptance-based approach, combined behavioral and acceptance-related components | Intervention: 1) Group meetings: - Self monitoring of weight - diet - exercise - stress management Control: none | Provider: Graduate students with behavioral weight loss experience Number: 8 Timing: weekly for 4-5 sessions, then monthly Duration: 75 minutes each | I: 16 weeks F: 12 months | N/A | 7.8 sessions completed on average |
| Lytle 2017 | Community College | Program planning approach; informed by ecological theories of health behavior, social cognitive theory and social network theory | Intervention:  1) 1 credit course focused on behaviors related to healthy weight maintenance; social network website encouraged self-monitoring, goal setting, and interaction between students  Control:  1) health assessments,  2) existing public health information on maintaining a healthy weight, and  3) information on health services offered on their school’s campus  Control: none | Provider: Study staff  Number: 12 classes Timing: 1 credit course (months 1-4), website access (months 1-24) Duration: 1 hour | I: 4 months F: 24 months | N/A | N/A |
| Metzgar 2016 | Community, Champagne-Urbana area | Social cognitive theory | Intervention:  1) group nutrition education sessions designed to increase self-efficacy, self-regulation, outcome expectations, and family and social support.  - Address energy balance through sustainable diet, exercise, and behavior modification Control: wait-list control | Provider:  1) Registered dietitians (RD): all female, practicing for <5 years 2) Counselors (CS): female, graduate teaching assistants non-nutrition programs Number: 24 Timing: weekly (months 1-4), monthly (months 5-12) Duration: 39.1 (±11.1) minutes; RD vs CS, 43.3 (±9.2) vs. 33.5 (±11.2) minutes,  **p < 0.01** | I: 12 months F: none | Engagement and content addressed were equal in groups. RDs were less likely to read from script, reinforce points, and provide specific scenarios than counselor. | On average (±SD), 2.9 (±1.6) participants attended each  session, and this did not differ between groups.  Weekly, monthly and overall compliance did not differ by group assignment. Compliance is defined as attendance at >85 % of education sessions (14/16 for weekly sessions, 7/8 for monthly sessions, 21/24 overall). 88.6 % (n = 39) of thosel enrolled after 16 weeks (n = 44) were deemed compliant with weekly attendance (RDG = 20; CSG = 19), and 81.5 % (n = 22) who completed the intervention (n = 27) were compliant with monthly attendance (RDG = 10; CSG = 12).  Overall, 88.5 % (n = 23) of participants that completed 12 month testing (n = 27) were compliant (RDG = 10; CSG = 13). |
| Valve 2013 | Vaccination centers (Finland) | Methods and principles of solutions-focused brief therapy | Intervention:  The LINDA solution-focused brief therapy intervention (goal setting, positive feedback) focused on:  - healthy physical activity  - dietary behaviors  - sleeping behaviors  Control:  Control group participants followed the HPV-008 study protocol, including counseling on sexual health and lifestyle counseling according to standard care in Finland. | Provider: Nurse Number: 3-5 meetings Timing: every 6 months Duration: 20 minutes | I: 1.5-2.5 years  F: none | N/A | I: Participants that received counseling on specific health behavior: Physical activity: 47% Dietary behaviors: 36% Sleeping behaviors: 20% Any target health behavior: 71% |
| Wing 2016 | Clinical setting (2 sites) | Self-regulation model, including feedback-based viewpoint on self-regulation | Intervention:  Behavior modification skills to implement prescribed changes  Small changes:  - decrease daily calories by 100,  - increase daily steps by 2000 Large changes:  - advised to lose 5-10 lbs first 8 weeks, - consume 1200-1800 calories  - exercise 250 min/week Control:  - daily self-weighing - information on healthy eating, physical activity, overview of small/large change strategies - access to study website with info on weight loss | Provider: Interventionists with master’s level backgrounds in nutrition, exercise physiology, or psychology and previous weight control experience Number: 10 in-person meetings, 2 online refresher campaigns Timing: 10 meetings in first 4 months, 4 week online refresher each year (total 2 years), monthly emails with feedback Duration: unknown | I: 4 months F: 3 years (average) | 100% of 20% recorded sessions presented appropriate behavioral content and distinguished the two active interventions | Average sessions attended (10 total sessions): Large change: 87.4%  Small change: 86%  Control: 100% attended 1 session |

Appendix 4: Effectiveness

| **First author, year** | **Retention rate (%) at program completion   Control vs. Intervention (% differential retention)** | **Compared dropouts** | **Significance of BMI and/or weight (kg) outcomes** | **Mediators/ moderators** | **Unintended consequences measures and results** | **Cost, Maintenance of program after completion of study** |
| --- | --- | --- | --- | --- | --- | --- |
| Bertz 2015 | Overall: 81%  C: 82.9%  I: 71.8% | No difference in dropout by intervention group. | BMI change at 12 month, mean (SD): C: 0.35 (1.40) I: -0.15 (1.23)  p = **0.033** | Moderator: gender | Rapid weight change;  Negative feeling affected by self-weighing (n=2) | None, Not reported |
| Biddle 2015 | Overall: 71%  C: 27%  I: 32% | Control group:  Non-completers tended to live in areas with higher multiple deprivation score (p=**0.01**) and tend to be unemployed (p=0.09) | BMI change at 12 month, mean (95% CI): C: -0.03 (-0.92, 0.31) I: -0.21 (-0.83, 0.40) p = 0.609 | None | None | None, Not reported |
| Greene 2012 | Overall: 66.7%  C: 70.3%*  I: 64.5%*  ***p<0.05** | Overall:  BMI/completer trend was not different between control and intervention group.  Control Group:  Completers were more likely to be, white, lower baseline BMI, desired to lose less weight, and had a higher eating competence than non-completers (70.3% vs 64.5%). | BMI, baseline to 15 month, mean (SE): C: 23.5 (0.19) - 23.9 (0.20) kg/m^2^ I: 23.3 (0.20) - 23.5 (0.21) kg/m^2^ p > 0.05 | Moderator: gender (interactions between group, gender, and time were assessed) | One male (.23%) and 12 females (1.7%) dropped below a normal weight status. | None, Not reported |
| Kattelmann 2014 | Overall: 59.4%  C: 60.3%  I: 58.4% | Overall:  More completers were female (70.4% vs 60.7%) and had never used cigarettes (71.7% vs 65.6%). | BMI baseline to 15 month, mean (SD): C: 24.2 (4.9) - 24.6 (4.9) kg/m^2^ I: 23.9 (3.9)- 24 (3.9) kg/m^2^ p = 0.5 | Moderator: gender | None | None, Not reported |
| Katterman, S. 2014 | Overall: 64%  C: 62.1%  I: 65.5% | Overall:  No difference in baseline measures between completers and non-completers | BMI change at 12 month: C: +0.34 kg/m^2^ I: -0.74 kg/m^2^ p = **0.008** | Mediator: Behavioral or acceptance-based variable changes from baseline to 6 weeks (no significant effect) | None | None, Not reported |
| Lytle 2017 | Overall: 84%  C: 82.5%  I: 83.5% | Overall:  There were statistically significant differences (p≤**0.05**) between the sample retained for the entire 24 months and the sample lost to follow-up by race/ethnicity (with those lost to follow-up more likely to be non-white) and by income (with those lost to follow-up more likely to have a higher income or report they didn’t know their income).  Women in the intervention group I were more likely to drop out as compared with women in the control group C (p = **0.05**) | BMI after 24 month, mean (SD): C: 26 kg/m^2^ (0.28) I: 26.2 kg/m^2^ (0.283) p= 0.699 | None | None | Tuition cost covered in intervention ($38,000), Not reported |
| Metzgar 2016 | Overall: 55%  C: 80.8%  Registered dietitian group (RGD): 42.3%  Counselor group (CSG): 55.2% | Women who completed the intervention (n = 48; mean ± SD, age: 33.4 ± 7.2 y; BW: 79.7 ± 19.7 kg; BMI: 29.5 ± 7.2 kg/m2) were significantly  older (P < **0.01)** as compared to women who did not complete the intervention (n = 33; mean ± SD, age: 28.4 ± 8.6 y; BW: 70.7 ± 17.0 kg; BMI: 25.6 ± 5.6 kg/m2); study  completers also were heavier as compared to women who did not complete the study, but this was not significant  (P > 0.01). | BMI baseline to 12 month, mean (SE) C: 29.3 (0.7) - 29.1 (0.8) kg/m^2^ RDs: 26.1 (0.5) - 26.6 (0.6) kg/m^2^ Counselor: 27.4 (0.4) - 27.8 (0.5) kg/m^2^ p > 0.05 | None | None | None, Not reported |
| Valve 2013 | Overall: 87.8%  C: 87.9%  I: 87.8% | Not reported | BMI change at 1.5-2.5 years, median (IQR): C: 0.51 (1.75)  I: 0.55 (1.59) p=0.996 | None | None | None, Not reported |
| Wing 2016 | Overall:  Baseline: 99%  4 months: 92%  1 year: 79%  2 years: 76% | Not reported | Weight change over average 3 years, mean (SE): C: 0.26 (0.22) kg Small changes (SC):  -0.56 (0.22) Large changes (LC):  -2.37 (0.22) p (C vs SC) = **0.018** p (C vs LC) **<0.001** p (SC vs LC) **<0.001** | Moderator: sex, age, and baseline weight (no significant effect) | Lost >20% baseline weight (n=7) | None, Not reported |

Appendix Table 5: Risk of bias, Cochrane judgements and reviewer comments

| **Author last name, year** | **Allocation concealment** | **Blinding of outcome assessors, weight/BMI outcome** | **Blinding of participants and personnel** | **Incomplete outcome data** | **Selective outcome reporting** | **Other sources of bias** |
| --- | --- | --- | --- | --- | --- | --- |
| Bertz 2015 | Unclear  There is no information about concealment of the allocation sequence and baseline differences between intervention groups appear to be compatible with chance. | Low  Outcome was recorded automatically from Wi-Fi scales. | Unclear  Unclear if CTM and C groups were blinded. Both groups received scales and the same set of information regarding risk of weight gain in young adulthood and strategies to maintain healthy weight. | Low  Intention to treat analysis - handling of missing data. | Unclear  All pre-determined outcomes are reported sufficiently. However, clinical trials registry reports the duration of this study as 3.5 years, the authors only report 1 year. | Unclear  Funding source - private donation |
| Biddle 2015 | Unclear  Randomized after baseline visit. | Low  Primary outcome assessed by wearable device. | High | High  High attrition at follow up visits and we underestimated dropout and non-compliance with the primary outcome measure. This led to large  amounts of missing data | Low  Authors report every outcome in a supplemental table. | Low |
| Greene 2012 | Unclear  Randomized after enrollment, no specific information. | Low  No information provided, however blinding of assessors unlikely to influence the outcome. | High  Participants cannot be blinded - unclear if personnel were blinded. | Control group was more likely to complete follow-up assessment. Completers had lower baseline BMI and waist circumference and desired to lose less weight than non-completers. | Unclear | Low |
| Kattelmann 2014 | Unclear  Baseline assessments were made after randomization. | Low  No information provided, however blinding unlikely to affect the outcome. | High  No information provided, but participants cannot be blinded to their randomization group. | Low  Missing outcome data not likely related to differences in baseline characteristics, no difference in completers and non-completers | Low  Authors reported all primary and secondary measures, including those with no significant difference (Tables 2 and 3). | Low |
| Katterman 2014 | Unclear  Randomized after enrollment | Low  No information provided, however blinding of assessors unlikely to influence the outcome. | High  Blinding is not conducive to this study type (of participants or personnel) | Low  Retention rates did not differ by condition or baseline characteristics. | Low  Measures described in methods reported in outcomes. | Low |
| Lytle 2017 | Unclear  Randomized after baseline assessment. | Low  No information provided, however blinding of assessors unlikely to influence the outcome. | High  Blinding is not conducive to this study type (of participants or personnel) | Low | Low  All outcomes reported. | Unclear  Differential retention by race/ethnicity and income. |
| Metzgar 2016 | Unclear  Participants were randomized after enrollment | Low  No information provided, however blinding of assessors unlikely to influence the outcome. | Low  Participants in the differing intervention groups were blinded to assignment until after the intervention was complete. | Low  Completers did not differ on BW or BMI, drop outs not related to study outcomes. | Low  All outcomes outlined in protocol have been reported. | Low |
| Valve 2013 | Unclear  Since randomization occurred on a site level of an existing trial, no way for participants to opt out based on their randomization status. | Low  No information provided, however blinding of assessors unlikely to influence the outcome. | High  Blinding not possible in this type of study - nurses and participants aware of lifestyle counseling. | Unclear  Retention similar in intervention and control arms. Limited mention of differences between completers and non-completers. | Low  Outcomes discussed in methods were reported. | Low  Cluster-randomized study design: no recruitment bias (clusters were randomized after recruitment), analysis appropriate for a cluster design |
| Wing 2016 | Unclear  Randomization occurred after enrollment and baseline assessment. | Low  All assessments were completed by masked staff members, who were centrally trained and certified. | High  All participants received newsletters and personalized feedback; however ability to blind participants/personnel is limited due to intervention design (face-to-face group meetings) Blinding is not conducive to this study design. | Unclear  Retention did not differ between groups but no details on if completers differed from non-completers. | Low  Outcomes described in method were reported | Unclear  Enrolled small portion of those screened. 609/3387 (5821-2434 ineligible) |
